# Early drotrecogin alpha (activated) administration in severe sepsis is associated with lower mortality: a retrospective analysis of the Canadian ENHANCE cohort

**DOI:** 10.1186/cc7893

**Published:** 2009-05-20

**Authors:** Richard V Hodder, Richard Hall, James A Russell, Harold N Fisher, Bobbie Lee

**Affiliations:** 1 Divisions of Pulmonary and Critical Care Medicine, University of Ottawa, The Ottawa Hospital, 1053 Carling Ave, Ottawa, ON, Canada, K1Y4E9; 2 Departments of Anesthesiology, Medicine and Pharmacology, Associate Professor of Surgery, Dalhousie University, The Queen Elizabeth II Health Sciences Centre, 1796 Summer St, Halifax, NS, Canada, B3H 3A7; 3 iCAPTURE Centre for Cardiovascular and Pulmonary Research, St. Paul's Hospital, 1081 Burrard St., Vancouver, BC, Canada, V6Z 1Y6; 4 Eli Lilly Canada Inc., 3650 Danforth Ave, Toronto, ON, Canada, M1N 2E8; 5 Eli Lilly Canada Inc., Eli Lilly Canada Inc., 3650 Danforth Ave, Toronto, ON, Canada, M1N 2E8

## Abstract

**Introduction:**

Early multimodal treatment of severe sepsis, including the use of drotrecogin alfa (activated) (DrotAA) when indicated, is considered essential for optimum outcome. However, predicting which infected patients will progress to severe sepsis and the need for aggressive intervention continues to be problematic. We therefore wished to explore whether there were any potential early markers that might predict improved survival in response to early use of DrotAA in patients with severe sepsis. In particular, in the dynamic setting of severe sepsis, we postulated that changes in markers reflecting evolving rather than baseline clinical status might guide therapy.

**Methods:**

Data on a cohort of 305 Canadian patients from the open label ENHANCE trial of DrotAA in severe sepsis was retrospectively analyzed to search for potential clinical predictors of outcome in severe sepsis. Patients received a 96-hour infusion of DrotAA and were followed for 28 days. The association between time to treatment and mortality within subgroups defined by dynamic changes in various potential markers was explored.

**Results:**

Mortality at 28 days was 22.6% and the variables of age, time to treatment, and early changes in serum creatinine and platelet count were identified by logistic regression as independent predictors of mortality. Across all age ranges, 28-day mortality was lower when DrotAA was administered within 24 hours of first sepsis-induced organ dysfunction compared to administration after 24 hours for both subgroups of patients defined by changes in platelet count and creatinine within the first day.

**Conclusions:**

These findings suggest that when indicated, treatment with DrotAA should be initiated as soon as possible, regardless of age.

**Trial Registration:**

**Previous trial registration number**: NCT00568893

## Introduction

Severe sepsis is a complex infection-induced syndrome associated with high morbidity and mortality. Although the case fatality rate of severe sepsis may be decreasing, it remains unacceptably high at 20 to 35% and because the incidence of severe sepsis is steadily increasing, the total number of deaths continues to increase [1-3].

In the setting of severe sepsis, early multi-modal, goal-directed therapy including early antimicrobial administration are recommended components of emergency treatment [4-9]; however, there is often underutilization and delay in the use of therapies with proven efficacy [4,5,10,11]. Although early changes in organ failure and even changes in clinical status within the first day are prognostic [12,13], clinicians commonly struggle to identify risk factors that might reliably predict progression of infection to severe sepsis, septic shock, and death, which could signal the need for early aggressive intervention [14,15].

Mortality from severe sepsis correlates with the number of organ dysfunctions[12]. One of the hallmarks of severe sepsis that leads to organ dysfunction is a profound disturbance of the microvascular circulation [16,17]. The response to infection is associated with a simultaneous activation of inflammation and the coagulation cascade, and suppression of fibrinolysis. This results in significant microvascular dysfunction and amplification of the pro-inflammatory and pro-coagulant processes in many tissues, which thereby augments organ dysfunction [6,16,18].

Drotrecogin alfa (activated) (DrotAA) is a form of recombinant human activated protein C (APC), which has been shown to significantly reduce mortality from severe sepsis and septic shock in patients with two or more organ failures [6,19]. DrotAA is recommended as part of a 'sepsis bundle' together with an early goal-directed approach to resuscitation [4,5,11,20].

Although the precise mechanism of clinical benefit of APC is uncertain, it may in part be derived from a modulating effect on microvascular inflammation and coagulation [21]. APC is present in concentrations ranging from 3 to 5 μg/mL in healthy adults and reduction of these concentrations below 50% of normal predisposes to thrombosis, predominantly in the venous system. When protein C becomes activated (APC) it has anti-thrombotic, pro-fibrinolytic and anti-inflammatory properties [16,20-22] that are important in combating microvascular coagulation and inflammation in sepsis. The mechanism of its benefit in these patients appears to be in part through direct interactions with the endothelium [21]. APC can inhibit endothelial cell apoptosis and also has a direct effect on endothelial cytoskeletal rearrangement that strengthens endothelial tight junctions [21]. Another direct mechanism of action of APC on the endothelium is modulation of the endothelial monolayer, leading to increased cell-cell contact and decreased permeability [23].

The ENHANCE (Extended Evaluation of Recombinant Human Activated Protein C) study was an open-label study (n = 2378) of DrotAA in severe sepsis, undertaken to accumulate further evidence for the efficacy and safety of DrotAA treatment in severe sepsis [6]. The study found that treatment within 24 hours from first organ failure was significantly associated with lower mortality and the authors concluded that "more effective use of drotrecogin alfa (activated) might be obtained by initiating therapy earlier".

Because there is also continuing uncertainty regarding the predictors of outcome in severe sepsis, the present study had two objectives: first, to attempt to identify early predictors of mortality evident within the first day; and second to further understand how these predictors may be associated with survival in subgroups of patients (i.e. prognostic value). These represent data that would be crucial to clinicians managing these complex cases.

Clinicians often categorize acutely ill patients as being 'stable', 'worsening', or 'improving', although there is no consensus on what is objectively meant by these terms. Nevertheless, classifying patients in this way might conceivably alter the approach to management, specifically the level of aggressiveness of treatment. For example, in the setting of severe sepsis, there is an emerging clinical consensus that merely stable portends a worse outcome than improving [12]. We therefore also wished to explore whether there was a relation between the patient's apparent clinical status defined in terms of dynamic variables (i.e. stable, worsening, or improving), the use of DrotAA and outcome.

To this end, the Canadian ENHANCE investigators used the Canadian cohort's database to conduct an exploratory retrospective analysis. This cohort represents a robust, homogeneous population with relatively uniform processes of care when compared with the international cohort, and is a suitable population with which to explore these hypotheses.

## Materials and methods

Eli Lilly granted permission to use data from the original ENHANCE trial for the current analysis. Institutional review board consent for this retrospective data review was not sought as the Informed Consent Document for the original ENHANCE trial included provisions for retrospective review. The first patient was enrolled in the international study in March 2001 and the final patients completed the follow-up period in January 2003.

Canadian sites had received institutional review board approval and appropriate informed consent was obtained from all patients. Data from all patients enrolled in the 21 Canadian sites of the multinational open-label ENHANCE trial [6] (25 countries, 361 sites, 2378 patients) were selected for this report. All patients (n = 305) received study drug infusion and were assessed at 28 days post-infusion.

All patients had known or suspected infection and manifested systemic inflammatory response syndrome (SIRS) defined by the presence of at least three of four SIRS criteria [24]. In addition, patients had one or more acute sepsis-induced organ dysfunctions (cardiovascular, respiratory, renal, hematologic, or metabolic acidosis) of 48 hours or less. Patients considered to be at high risk for clinically important bleeding, or with known hypocoagulable or hypercoagulable conditions were excluded. A full description of the inclusion and exclusion criteria have been previously published [19].

DrotAA (Xigris^©^, Eli Lilly and Co., Indianapolis, IN, USA) was infused in an unblinded fashion in all patients at a dose of 24 μg/kg/hour for 96 hours within 48 hours of the diagnosis of the first organ dysfunction. Standard supportive care was provided until discharge.

The primary endpoint was all-cause mortality at 28 days. From the 48-hour period prior to infusion until day 28 post-infusion, organ function, markers of disease severity, incidence and type of infection, and other laboratory tests were assessed. Throughout the 28-day period, vital signs, infection status, transfusion status, and adverse events were recorded.

This was a retrospective exploratory analysis rather than a prospective trial, and one of our objectives was to explore whether there was a relation between the patient's dynamic clinical status (i.e. stable, worsening, or improving), the use of DrotAA, and outcome. However, because different physicians may view stable as either 'good' (i.e. same category as improving) or 'bad' (i.e. same category as worsening), we chose to consider a single composite clinical status category, namely 'stable/worsening'. Furthermore, because these potentially prognostic terms are only loosely defined, we attempted to objectify the definitions for the present analysis in terms of changes in commonly measurable clinical parameters. Following the results of multivariate logistic regression while adjusting for multiple co-variates, variables identified to be statistically significant in the bivariate analysis were fitted into a logistic regression model. Based on this analysis, we chose to define the term stable/worsening in terms of dynamic changes from baseline in serum creatinine and platelet counts. Additional details on this methodology are described in the results section below.

### Statistical analysis

Descriptive statistics, including mean, standard deviation, count, and percentages, were performed on baseline characteristics and disease severity markers for the Canadian sample. Baseline demographics, disease severity, and clinical variables that were associated with mortality were compared in a bivariate analysis between survivors and non-survivors. One-sided Wilcoxon rank sums test were used for continuous variables and Chi-square or Fisher's exact tests were used for categorical variables.

We wished also to explore whether there was a relation between the patient's apparent clinical status (ie. stable, worsening, or improving), the use of DrotAA, and outcome. However, because there is no consensus on these clinical definitions of stability and because different physicians view stable as either good (i.e. same category as improving) or bad (same category as worsening), we chose to consider a single composite clinical status category, namely stable or worsening, as there is an emerging clinical consensus that stable portends a worse outcome than improving [12].

Separate analyses were performed for time to treatment and mortality rate on the stable/worsening clinical status subgroup. Twenty-eight-day mortality was compared between patients who received early treatment (defined as less than 24 hours from first sepsis-induced organ dysfunction to start of infusion of DrotAA) and those who received late treatment (24 hours or more to start of infusion of DrotAA). The analysis was repeated for each category of clinical status using Cochran-Mantel-Haenszel statistics. The Breslow-Day test was used to examine time to treatment and age interactions.

To identify possible predictors of mortality, multivariate logistic regression was used while adjusting for multiple co-variates. Variables identified to be statistically significant in the bivariate analysis were fitted into a logistic regression model. Correlations between the variables that were retained in the model using the stepwise method with a significance level of 0.05 were examined for multicollinearity.

Odds ratio estimates, 95% confidence intervals, and *P *values were generated. A few variables were rescaled so that the odds ratio estimates reflected incremental changes at respective intervals. Although the variables remained as continuous variables, the variables for change from baseline to day 1 in creatinine concentration and platelet count were divided by 50 for convenience in reporting.

We also sought to explore whether the timing by clinically important intervals would stand as an independent predictor of survival. Following extensive exploratory analysis, we identified that a six hour cutoff increment appeared to make a difference in terms of survival, and so the variable for time from first sepsis-induced organ dysfunction to start of study drug was divided by six in order to determine if there were differences in outcome by six-hour intervals from onset of organ dysfunction to treatment. Age was classified into four groups (≤49, 50 to 64, 65 to 74, and ≥75 years of age) to aid in clinical interpretation.

All statistical tests were performed at an alpha level of 0.05. There was no adjustment for multiple comparisons.

## Results

### Baseline characteristics and outcome

The Canadian cohort from the international ENHANCE study contained 305 patients enrolled at 21 sites across Canada (see list of investigators), 141 (46%) of whom received DrotAA within the first 24 hours and 164 (54%) of whom received DrotAA after 24 hours. Patient demographics and disease severity are summarized in Table 1. Half of the patients (50.2%) had organ dysfunction in each of the cardiovascular, renal, and respiratory systems, indicating that this was a severely ill cohort.

**Table 1:** Patient baseline characteristics and disease severity markers for patients receiving DrotAA

**Variable**	**Canadian sites (n = 305)**
Gender, % male	57.7

Mean age, years (SD)	56.9 (17.2)

Mean APACHE II (SD)	25.3 (7.8)

Mean number of organ dysfunctions (SD)	3.1 (1.2)

Mean SOFA score (SD)	10.9 (3.6)

Vasopressor use	81.6%

Mechanical ventilation	90.5%

APACHE II = Acute Physiology and Chronic Health Evaluation II; SD = standard deviation; SOFA = Sequential Organ Failure Assessment.

### 28-day mortality analysis

Exploratory analysis of the differences between survivors (n = 236) and non-survivors (n = 69) for the Canadian cohort revealed the significant or clinically important variables listed in Table 2. These variables were then used in a bivariate analysis of survivors and non-survivors and the results are presented in Table 3. Age, an increase in serum creatinine from baseline to day 1, a decrease in platelet count from baseline to day 1, the number of organ dysfunctions at baseline, and pre-infusion Acute Physiology and Chronic Health Evaluation (APACHE) II score were significantly associated with 28-day mortality. Thus, non-survivors at 28 days were on average older, had more severe disease at baseline and had greater early progression of renal and hematological dysfunction.

**Table 2:** Variables used in bivariate analysis

Age (years)
Pre-treatment APACHE II score

Number and type of organ dysfunctions at baseline

Time (hours) from sepsis-induced organ dysfunction to DrotAA administration

Baseline vasopressor status (on vasopressors or not on vasopressors)

Baseline ventilator status (ventilated or not ventilated)

Baseline laboratory values and changes over time:
• platelet count
• serum creatinine
• protein C
• prothrombin time

Baseline and Day 1 SOFA scores

Study site participation in PROWESS [19] (participated in PROWESS or did not participate in PROWESS)

APACHE = Acute Physiologic and Chronic Health Evaluation; DrotAA = Drotrecogin alfa activated; PROWESS = Recombinant human activated protein C worldwide evaluation in severe sepsis; SOFA = Sequential Organ Failure Assessment.

**Table 3:** Variables associated with 28-day mortality in Canadian sites in ENHANCE

	**Survivors** Mean (SD)	**Non-survivors** Mean (SD)	*P *value*
Age – years	55.4 (17.4)	62.3 (15.4)	0.0014

Change in serum creatinine (μmol/L), pre-infusion** to day 1	2.8 (85.7)	31.0 (66.0)	0.0011

Change in platelet count (× 10^9^/L) from pre-infusion to day 1	-10.2 (47.7)	-27.6 (59.0)	0.0052

Number of organ dysfunctions per patient at baseline	3.0 (1.16)	3.4 (1.088)	0.0073

Baseline APACHE II score	24.7 (7.65)	27.4 (8.153)	0.0100

Time to treatment – hours	26.1 (11.3)	27.6 (14.0)	0.2447

Time to treatment <24 hours	80.9%	19.1%	0.1788

Time to treatment ≥24 hours	74.4%	25.6%	

* *P *value is based on a one-sided Wilcoxon rank sums test; ** lowest creatinine recorded pre-infusion.

APACHE = Acute Physiologic and Chronic Health Evaluation; SD = standard deviation.

Although time to treatment was not statistically significant in this bivariate analysis, the variable was retained in subsequent analyses based on results from the full ENHANCE trial, which demonstrated that time to treatment within 24 hours from first organ failure was significantly associated with lower mortality. In addition, we felt that timing was an important clinical issue, and that there might be other factors confounding the association between mortality and treatment.

### Effect of early versus late treatment with DrotAA

A number of significant mortality predictors were identified by the bivariate analysis: age, baseline Sequential Organ Failure Assessment (SOFA) respiratory score, baseline prothrombin time, number of organ failures at baseline, pre-infusion APACHE II score, baseline SOFA cardiovascular score, change in platelets, and change in creatinine.

The interaction between age and early versus late treatment with DrotAA was examined in the predefined clinical subgroup stable or worsening (n = 242). For these patients, early treatment with DrotAA was associated with lower mortality regardless of age (*P *= 0.0409; Table 4).

**Table 4:** 28-Day mortality and early versus late treatment with DrotAA in patients with clinically stable or worsening status*

**Age years**	**DrotAA <24 hours**		**DrotAA ≥24 hours**		***P *value**
	**n/N**	**% Mortality**	**n/N**	**% Mortality**	

≤49	3/34	8.80	6/44	13.60	0.0409
	
50 to 64	3/27	11.10	12/43	27.90	
	
65 to 74	5/24	20.80	9/25	36.00	
	
≥75	6/22	27.27	8/23	34.78	

*P *value was based on Cochran-Mantel-Haenszel test adjusted for age (Breslow-Day *P *> 0.05). * Defined by first day change in serum creatinine or platelet count.

DrotAA = Drotrecogin alfa activated; n = number of patients who died by day 28; N = total number of patients.

Table 5 displays the odds ratio estimates of dying for those variables found to be significant independent predictors of 28-day mortality. The results suggest that when treated with DrotAA, patients aged 49 years and younger have lower odds of mortality than those 65 years and older (odds ratio: 0.25 to 0.27), and that each six-hour delay in starting DrotAA after the first sepsis-induced organ dysfunction was associated with a 23% higher odds of mortality (odds ratio: 1.23). A rise in serum creatinine of 50 μmol/L from baseline to day 1 increased the odds of mortality by 32% (odds ratio: 1.32), whereas an increase in platelet count of 50 × 10^9^/L from baseline to day 1 decreased the odds of mortality by 34% (odds ratio: 0.66).

**Table 5:** Odds ratio estimates of 28-day mortality for patients receiving DrotAA

**Variable**	**Odds ratio estimate (95% CI)**	***P *value**
**Age, years**		

≤49 vs 50 to 64	0.524 (0.2203 to 1.2461)	0.144

≤49 vs 65 to 74	0.251 (0.102 to 0.617)	0.003*

≤49 vs ≥75	0.269 (0.106 to 0.678)	0.005*

50 to 64 vs 65 to 74	0.479 (0.2146 to 1.0676)	0.072

50 to 64 vs ≥75	0.513 (0.2226 to 1.1802)	0.116

65 to 74 vs ≥75	1.071(0.4524 to 2.5346)	0.876

**Time to treatment**		

Delay in time from first sepsis-induced organ dysfunction to start of study drug (six-hour intervals)	1.231 (1.053 to 1.439)	0.009*

**Laboratory values**		

Increase in creatinine from baseline to day 1 (50 μmol/L intervals)	1.317 (1.033 to 1.679)	0.026*

Increase in platelets from baseline to day 1 (50 × 10^9^/L unit intervals)	0.658 (0.442 to 0.978)	0.039*

* statistically significant. CI = confidence interval; DrotAA = Drotrecogin alfa activated

### Safety

Bleeding was the only serious adverse event related to DrotAA administration. Serious bleeding events were defined as life-threatening or disabling events, intracranial hemorrhage, or bleeding that required transfusions for two consecutive days. A descriptive analysis comparing the Canadian and non-Canadian cohorts revealed that in the Canadian cohort, bleeding events were relatively few and less frequent compared with non-Canadian sites. For example, during the 96-hour infusion period, 2% of Canadian patients had serious hemorrhage (versus 3.8% of non-Canadian patients), and 0.3% had intracranial hemorrhage (versus 0.7% of non-Canadian patients). In the 28-day study period (which included the 96-hour infusion), serious bleeds occurred in 3.9% of Canadian patients (6.9% of non-Canadian patients).

## Discussion

In severe sepsis there is uncertainty as to which clinical predictors may be valuable in determining whether a patient's clinical status is stable or not. Our study suggests that several readily available variables may be potential early clinical predictors of outcome, and that, when indicated, early rather than late administration of DrotAA is associated with improved outcome in severe sepsis. This retrospective analysis suggests that in combination with other treatment modalities, when indicated, DrotAA administered within 24 hours of the first sepsis-induced organ dysfunction, may result in significant survival benefits for patients across the age spectrum studied. This holds true for patients demonstrating a stable or worsening clinical status, at least as defined by dynamic changes in serum creatinine level and in platelet count.

### Mortality predictors in severe sepsis

Our initial analysis revealed that in patients administered DrotAA, two baseline measures of severity – mean number of organ dysfunctions and APACHE II score – and two dynamic laboratory measures – serum creatinine and platelet count – were associated with 28-day mortality. Following a logistic regression for independent predictors, only a subset of the variables remained statistically significant: age, first day change in serum creatinine, first day change in platelet count, and time to treatment.

Most clinical studies have utilized 'static' characteristics obtained at baseline to predict mortality. The finding in the current analysis that early changes in serum creatinine and platelet count were independent predictors of mortality has direct clinical implications. In the rapidly changing environment of severe sepsis, it is helpful to identify predictors that reflect evolving rather than simply baseline status. The idea that identification of early dynamic predictors of mortality could be very useful in guiding therapy has recently been suggested from an analysis of dynamic coagulation changes in sepsis [18]. Dhainaut and colleagues found that continued or worsening coagulopathy during the first day of severe sepsis was associated with new organ failure and increased mortality at 28 days [18]. Data from an integrated sepsis database also provides evidence that serum creatinine change within the first post-baseline day is predictive of outcome [8]. Other outcome predictors have been identified in severe sepsis [7,25]. Micek and colleagues observed that the number of organ dysfunctions (adjusted odds ratio: 2.30) and inappropriate antimicrobial treatments (adjusted odds ratio: 15.5) were independent predictors of mortality [7]. In addition, Johnston found that baseline platelet counts less than 80 × 10^9^/L carried an odds ratio of death of 2.05 and a baseline prothrombin time greater than 30 was associated with an odds ratio of death of 2.88 in severe sepsis [25].

### Time to treatment with DrotAA and 28-day mortality

We observed that the risk of mortality based on odds ratios increased by 23% with each six-hour delay in receiving DrotAA. Because this was seen only in the retrospective subgroup analysis, it could be merely reflective of chance. On the other hand, there could be subsets of patients who will benefit more from earlier treatment. This possibility is supported by others who also found value in early treatment [4,26]. In an analysis of the full ENHANCE sample, the adjusted odds of death were 21.8% higher for patients treated later than 24 hours after the first sepsis-induced organ dysfunction, compared with treatment within 24 hours, although the association was not statistically significant when adjusted for age [6]. In the current analysis, when a further breakdown of age was used and the time to treatment effect was based on odds ratios for death, time to treatment remained statistically significant after adjusting for age. However, as this is a *post hoc*, exploratory analysis, this should be considered as hypothesis-generating only, and interpretation should be made with caution.

Others have also observed value in early treatment of severe sepsis with DrotAA. A recent meta-analysis of five clinical trials using DrotAA found that patients treated within 24 hours of first sepsis-induced organ dysfunction had significantly higher 28-day survival compared with those treated later than 24 hours (76.4% versus 73.5%) [27]. These investigators also found that in their adjusted model, logistic regression analysis suggested that treatment with DrotAA within 24 hours of organ dysfunction was associated with lower odds of death (23%), compared with treatment after 24 hours. In a prospective study of mortality predictors by Micek and colleagues, the time to treatment with DrotAA in survivors was significantly earlier (16.9 hours, standard deviation (SD) 11.9 hours) compared with non-survivors (25.5 hours, SD 28.0 hours) [7]. Most recently, Kanji and colleagues, in a Canadian observational study, also found that early treatment with DrotAA (within 12 hours of first organ dysfunction) was associated with a lower risk of death [28].

### Factors associated with delayed aggressive intervention in severe sepsis

International guidelines for the management of severe sepsis suggest the use of DrotAA for patients at high risk of death [13]. Furthermore, results from the current exploratory evaluation suggest that treatment with DrotAA should be initiated as soon as possible in all patients with sepsis-induced organ dysfunction who meet criteria for its administration. However, delays in initiating treatment with DrotAA in Canada are common – in a registry of 4087 Canadian intensive care patients (1269 with severe sepsis), the average time to treatment with DrotAA after identification of organ dysfunction was 1.3 days [29]. Others have highlighted potentially harmful consequences of delayed therapy with DrotAA [27]. A meta-analysis in 4459 patients demonstrated that delays in treatment were directly correlated with the number of organ dysfunctions, the need for mechanical ventilation or vasopressors, or recent surgery [27]. In addition, that analysis found an inverse relationship between baseline APACHE II score and time to treatment [27]. Some practice guidelines [20] recommend that DrotAA be withheld when the clinical course is uncertain, which may further contribute to worsening clinical status and delays in therapy. Some clinicians may thus wait for an APACHE II score to exceed 25 before starting DrotAA, as suggested by the Food and Drug Administration [25], despite the fact that APACHE scoring has been validated as a static baseline measure only in populations and not in individual patients, and despite the fact that multiple organ dysfunction may be improving or worsening in the face of no or minor changes in APACHE score. Inherent in such delays in escalating therapy may be the impression held by some that apparent clinical stability is a sign that the patient with severe sepsis may soon begin to improve with the current level of therapy.

The concept of a false sense of security due to apparent clinical stability may be particularly important when one considers that the clinician's impression of what constitutes clinical stability is usually based on 'macroscopic' observations (e.g. blood pressure, urine output, need for vasopressors etc), whereas the underlying pathophysiology of severe sepsis is best defined in terms of a pathologic microcirculation [17,30]. For example, it has been demonstrated that microvascular dysfunction can persist in at least 40% of patients who appear clinically stable [11]. Apparent clinical stability may thus not reflect underlying pathophysiology nor predict survival in severe sepsis. Deficits in microvascular function are present at the earliest stages of sepsis and improved tissue oxygen delivery relative to tissue oxygen demand may prevent progression of sepsis [22]. Treatment addressing 'hidden' microvascular dysfunction may be one potential explanation of why clinically stable patients appear to benefit from early treatment with DrotAA [31]. Treatment with DrotAA may reduce microvascular inflammation and coagulation, and thus improve function and tissue oxygenation.

Ideally therefore, aggressive medical intervention for the reversal of organ dysfunction should be initiated by addressing microvascular dysfunction, inflammatory response, and coagulopathy. However, escalation of therapy is usually based on clinical status and is thus often reserved for patients who are overtly deteriorating or at immediate risk of death. Clinicians may therefore hold off intervention with DrotAA or other aggressive resuscitation measures if they interpret that a patient is 'stable', and may wait a significant amount of time in order to determine a 'clear' increased or increasing risk of death.

On the other hand, because many clinicians increasingly view clinical stability in severe sepsis as having a similar outcome to worsening clinical status, we attempted to explore the impact of DrotAA therapy in patients who were defined to be clinically stable or worsening, based on changes in serum creatinine and platelet count. Although the intent was to perform the analysis in a way that mimicked actual clinical practice, combining stable patients with the worsening group does introduce a potential limitation, particularly because these definitions were determined *post hoc*. For example, it is possible that some patients who were started on DrotAA during the first 24 hours after presentation may have received drug before changes in creatinine and or platelet counts were observed. It should be emphasized, therefore, that the present analysis is exploratory for the purpose of hypothesis generation, and that we cannot at present recommend that serial changes in creatinine or platelet count be used as a guide as to whether or not or when DrotAA should be given. In addition, although an attempt was made to mimic 'real world' clinical decision-making, stable patients made up the largest subgroup (n = 162) as defined by measures of stability in serum creatinine and platelet count. Inclusion of these patients with worsening clinical status as defined may have biased the results. Nevertheless, because delays in therapy with DrotAA are associated with increased mortality, we believe that the concept of apparent clinical stability as a guide to the aggressiveness of therapy for severe sepsis should be validated in a larger cohort.

This retrospective subset analysis of the open-label ENHANCE trial has a number of other limitations because of the *post hoc *and subgroup analyses. A sample size of 305 homogeneous patients may have biased the results because of a lack of power. Furthermore, predictor variables were collected during the administration of DrotAA, which may confound the prognostic validity of mortality predictors with the effects of treatment with DrotAA. In addition, the observed improvement in survival associated with early use of DrotAA may have been a marker for patients who received earlier and more aggressive resuscitation in the first place (i.e. early goal-directed therapy [11]). An analysis of the original global ENHANCE data suggests that this is unlikely, as well as the fact that this was a relatively new concept during the enrolment period. In the global ENHANCE study, patients treated later (>24 hours) were more likely to be male and older and to have had recent surgery. Patients treated later most likely had more severe disease as suggested by a greater need for vasopressors and mechanical ventilation, and a greater number of organ dysfunctions, and total SOFA score [6]. Patients treated earlier had significantly lower 28-day all-cause mortality (22.9%) than those treated later (27.4%) [6].

## Conclusions

This exploratory analysis provides further evidence for two hypotheses. First, that there are readily available early clinical predictors of outcome that may be valuable in assessing risk in the dynamic setting in severe sepsis. Indeed, we observed that apparent stability of serum creatinine and platelet counts was associated with significantly increased 28-day mortality. Second, early treatment with DrotAA (within 24 hours of onset of sepsis-associated organ dysfunction) may confer a survival benefit compared with later treatment. Waiting until clear signs of clinical deterioration are present before initiating treatments for severe sepsis may represent a lost opportunity for improvement and confer a worse prognosis. These results should be considered preliminary and hypothesis generating and require validation using a larger prospective sample. Specifically, this exploratory analysis suggests that future clinical trials in sepsis should include provision for prospectively collecting data during the first 24 hours on early dynamic changes in easily measured variables that might help develop a reliable predictive index to help identify patients most likely to benefit from the most aggressive therapies available.

## Key messages

• Early clinical predictors of outcome in severe sepsis exist and should be sought.

• Stability of serum creatinine and or platelet numbers in early sepsis may be a negative predictor of outcome and should signal the need for re-assessment of management.

• Treatment with DrotAA within 24 hours of the diagnosis severe sepsis may be associated with improved outcome.

## Abbreviations

APACHE: Acute Physiologic and Chronic Health Evaluation; APC: activated protein C; DrotAA: Drotrecogin alfa activated; ENHANCE: Extended Evaluation of Recombinant Human Activated Protein C; SD: standard deviation; SIRS: systemic inflammatory response syndrome; SOFA: Sequential Organ Failure Assessment.

## Competing interests

Eli Lilly and Company sponsored the international ENHANCE trial and contributed to the publication costs of this manuscript. RVH and RH have received consulting and speaker reimbursements from a number of industry groups including Eli Lilly Canada. RVH, RH, and JAR have received investigator payments for the conduct of the ENHANCE trial, but have no financial interests in Eli Lilly or any of its products. JAR reports receiving consulting fees from Ferring, which manufactures vasopressin, and from Sirius Genomics Inc. JAR also reports receiving grant support from Sirius Genomics, Novartis, and Eli Lilly. HNF is an employee of Eli Lilly Canada and BL was an employee of Eli Lilly Canada at the time of the research and manuscript preparation. HNF holds shares in Eli Lilly Canada. JAR reports holding stock in Sirius Genomics Incorporated, which has submitted patents owned by the University of British Columbia and licensed to Sirius Genomics, that are related to the genetics of vasopressin and protein C. The University of British Columbia has also submitted a patent related to the use of vasopressin in septic shock. JAR reports being an inventor on these patents.

## Authors' contributions

RVH, RH, and JAR were clinical investigators in the ENHANCE trial and have had direct input into the drafting, revisions, and final approval of this manuscript. BL was responsible for statistical analysis, and HF had direct input into the drafting, analysis, and revisions of the manuscript.
